# Tumor-Stroma Ratio Is an Independent Prognostic Factor for Distant Metastasis in Squamous Cell Lung Cancer Following Resection

**DOI:** 10.1155/carj/9963742

**Published:** 2025-08-06

**Authors:** Fuman Wang, Yue Zhang, Dawei Li, Yifan Chi

**Affiliations:** ^1^Medical College of Qingdao University, Ningxia Road 308#, Qingdao 266000, China; ^2^Department of Cardiac Surgery, Haici Hospital of Qingdao University, Renmin Road 4#, Qingdao 266000, China; ^3^The First Clinical Medical College of Shandong University of Chinese Medicine, 16369#, Jingshi Road, Jinan 250012, China; ^4^Shandong Mental Health Center of Shandong University, Wenhuaxi Road 49#, Jinan 250012, China; ^5^Department of Urology, Qilu Hospital of Shandong University, Wenhuaxi Road 107#, Jinan 250012, China

**Keywords:** distant metastasis, squamous cell lung carcinoma, tumor-stroma ratio

## Abstract

Cancer distant metastasis is one of the main causes of cancer progression and difficulty in treatment (Rossi et al., 2020). This abstract aims to summarize the significance of tumor-stroma ratio (TSR) as a prognostic factor in the development of distant metastasis in squamous cell lung cancer (SQCLC) patients. The TSR has recently been recognized as a novel and independent prognostic parameter for a variety of solid tumor types (Lu et al., 2023). A total of 86 patients with SQCLC who had undergone surgery were included in the present study. Two independent observers visually identified TSR on hematoxylin and eosin (H&E)–stained pathological histologic sections. Patients were separated into two groups: stroma-rich, with a ratio of stroma as > 50%, and stroma-poor, with a ratio of stroma as ≤ 50%, which included a total of 36 and 50 patients, respectively. In the current study, the overall survival and no distant metastasis survival of patients in the stroma-poor group were improved compared with the stroma-rich group, and the overall risk of patients in the stroma-poor group was reduced compared with the stroma-rich group (*p* < 0.05). In the multivariable analyses, the TSR was recognized as an important prognostic indicator for overall survival (HR = 2.41; *p* < 0.001) and no distant metastasis survival (HR = 2.27; *p* < 0.001). The study revealed that in patients with SQCLC, stroma-rich tumors were associated with a shorter distant metastasis-free interval and poorer prognosis compared to stroma-poor tumors. These findings suggest that the TSR may serve as a novel prognostic indicator for predicting distant metastasis in SQCLC.

## 1. Introduction

Tumor microenvironment plays a critical role in the development and progression of various cancers, including squamous cell lung cancer (SQCLC). The tumor microenvironment consists of a complex network of noncancerous cells, extracellular matrix (ECM), blood vessels, and various signaling molecules [1]. It exerts significant influence on tumor behavior, including metastasis. Understanding the impact of tumor-stroma ratio (TSR) on distant metastasis in SQCLC is essential for developing effective treatment strategies and improving patient outcomes. SQCLC constitutes 20%–30% of non-small-cell lung cancer (NSCLC) cases, with 5-year survival rates below 15% in metastatic disease due to limited targeted therapies [2]. The absence of biomarkers for metastasis risk stratification underscores the need to explore TSR as a practical histopathological tool [2].

The TSR, defined as the proportion of stromal tissue to tumor tissue within a tumor specimen, has emerged as a potential prognostic factor in several cancer types [3]. In gastrointestinal cancer, the TSR has been found to be associated with tumor aggressiveness and patient survival [4]. Relevant studies suggest that high stromal composition may be associated with an increased risk of distant metastases of SQCLC and poor prognosis [5].

One key component of the tumor stroma in SQCLC is the ECM, which provides structural support to tumor cells and influences their behavior [1]. The ECM is composed of various proteins, including collagen, fibronectin, and laminin, which interact with cell surface receptors and modulate cellular processes such as migration, invasion, and angiogenesis. Alterations in the composition and organization of the ECM can promote tumor cell invasion and metastasis [6].

Moreover, the stromal cells within the tumor microenvironment, such as cancer-associated fibroblasts (CAFs) and immune cells, play crucial roles in tumor progression and metastasis [7]. CAFs, in particular, have been shown to promote tumor cell invasion and metastasis through the secretion of growth factors and cytokines. Immune cells, on the other hand, can have both pro- and antitumorigenic effects, with certain subsets facilitating metastasis and others suppressing it [8]. The balance between these diverse cell populations within the tumor stroma can determine the outcome of distant metastasis in SQCLC.

Furthermore, recent evidence suggests that the tumor stroma also influences the response to therapy [9]. The altered ECM composition and increased presence of immune cells within the tumor stroma can impact drug delivery and the efficacy of treatment modalities [10]. Therefore, understanding the role of TSR in modulating treatment response is of great clinical importance.

This study aims to investigate the potential clinical significance of TSR in resectable SQCLC, with a primary focus on evaluating the prognostic value of TSR for predicting distant metastasis and overall survival (OS) in patients.

## 2. Materials and Methods

### 2.1. Study Population

**Figure 1:**
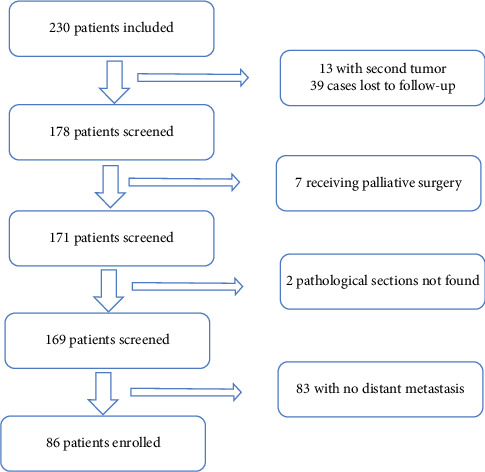
Screening chart for 230 patients who underwent squamous cell lung carcinoma surgical resection at Qilu Hospital of Shandong University.

This study is a retrospective study that included 86 patients with SQCLC who underwent complete surgery at Qilu Hospital of Shandong University from January 2015 to December 2018. Clinical data were obtained from patients' records, including gender, age, smoking history, tumor size, histology, differentiation degree, and pathological staging. In addition, hematoxylin and eosin (H&E)–stained slides of tumor tissues were collected by the Department of Pathobiology. Written consent was obtained from all patients before using tumor tissues, and the protocol was approved by the Ethics Committee of Qilu Hospital of Shandong University. The inclusion criteria for patients participating in this study were as follows: (i) patients who underwent successful surgical treatment, including curative surgery and systemic lymphadenectomy; complete surgery was defined as R0 resection via lobectomy (78%) or pneumonectomy (22%), based on tumor size and pulmonary function; metastasis risk was not a surgical decision factor [11]; (ii) patients with histologically confirmed SQCLC; (iii) patients with evidence of metabolic abnormalities but not detected through preoperative examinations, including positron emission tomography (PET) and CT; and (iv) patients who developed distant metastasis after surgery. The exclusion criteria were as follows: (i) patients with a second primary tumor; (ii) patients who underwent palliative surgery; (iii) patients who received neoadjuvant therapy, including radiation therapy, chemotherapy, or chemoradiotherapy; (iv) patients without complete follow-up data; and (v) patients who died within 30 days after surgery. A total of 86 patients were included in this study (Figure 1).

### 2.2. Staining and Evaluation

**Figure 2:**
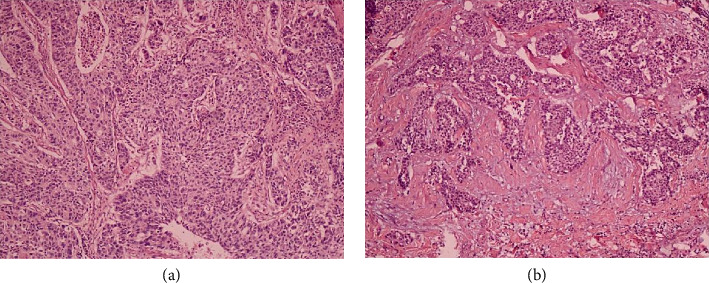
Hematoxylin and eosin–stained sections (5 μm) of squamous cell lung carcinoma (original magnification, × 100). (a) The stroma-poor squamous cell lung carcinoma group (stroma ratio < 50%). (b) The stroma-rich squamous cell lung carcinoma group (stroma ratio ≥ 50%).

Specimens were obtained from pathological archives for histopathological examination, using 5 um paraffin-embedded tissue sections stained with H&E. In general, areas with abundant stroma in tumor heterogeneity are considered to have a poorer prognostic value and are therefore considered determinative. Typically, stroma-rich areas are found near the deepest infiltrating point under a 4x objective lens (40x total magnification) and are further evaluated. Subsequently, microscopic fields that simultaneously display stroma and tumor are selected, with tumor cells being displayed on all sides and scored using a 10x objective lens (100x total magnification). Evaluation is based on the analysis of at least one microscopical field. This estimation is then recorded as the TSR. According to this protocol, tumor sections are independently evaluated by two researchers. In the case of discordant opinions between the two observers, the decision of a third pathologist plays a decisive role. TSR was scored per van Pelt et al. [11]: stroma-high (> 50%) and stroma-low (≤ 50%) [12]. Microscopical areas were selected for TSR quantification from stroma-rich and stroma-poor tumors and are indicated in Figure 2.

### 2.3. Follow-Up

**Figure 3:**
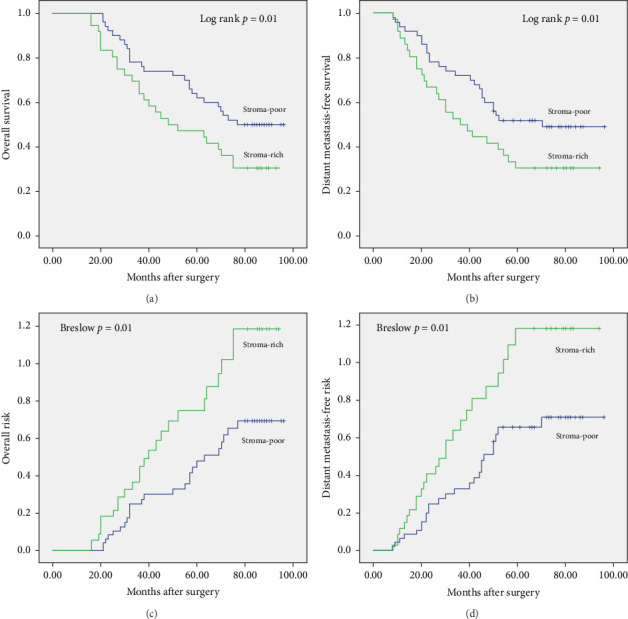
Kaplan–Meier survival and risk curves (stroma-poor vs. stroma-rich) for all 86 patients with squamous cell lung cancer. Overall survival (a) and survival without distant metastasis (b) between the two groups were statistically significant. The total risk (c) and the risk of no distant transfer (d) were also statistically significant.

Follow-up information was collected until December 2023 or until the patient's death. Follow-up duration varied (median 69 months; range 16–96).  Figure 3 truncates data at 100 months for uniformity. All patients underwent a complete medical history and physical examination every 3 months during the first 2 years after surgery, and then every 6 months thereafter. Medical imaging was performed during the follow-up period, including chest and abdominal CT scans, brain MRI, and PET. OS was defined as the time from the date of surgery to the end of patient follow-up or death. No distant metastasis survival was defined as the interval between the date of surgery and the occurrence of distant metastasis, based on the first occurrence.

### 2.4. Statistical Analysis

Statistical analysis software Version 24.0 (SPSS Incorporation, Chicago, IL, United States of America) was used to carry out statistical analysis. A *χ*^2^ examination was used to determine the difference between TSR and other clinicopathological characteristics, as was Fisher's accurate test. OS, no distant metastasis survival, and no distant metastasis risk curves were plotted using Kaplan–Meier survival analysis. While the survival curves were contrasted with log-rank examinations, the risk curves were contrasted with Breslow examinations. Cohen's *κ* coefficient was used to analyze the reliability of the pathologists. HR and 95% CI were analyzed to determine OS and no distant metastasis survival using single-variable and multivariable Cox regression patterns. *p* < 0.05 was considered to indicate a statistically significant difference.

## 3. Results

### 3.1. Clinicopathological Features

A total of 86 patients (76 men and 10 women) were included in the current study. The median age of the patients was 61 years (range, 41–79) at the date of the operation. The median follow-up period was 69 (range, 16–96) months. The pathological and therapeutic features of patients are presented in Table 1.

A total of 86 sick people were divided into two groups: a stroma-poor tumor group (TSR ≤ 50%; *n* = 50) and a stroma-rich tumor group (TSR > 50%; *n* = 36). The two groups were compared using a *χ*^2^ examination and Fisher's accurate test. Table 1 indicates that gender, age, smoking history, drinking history, differentiation class, adjuvant treatment, and pathological tumor-node-metastasis stage of the patients were not significantly associated with TSR.

### 3.2. TSR in Squamous Cell Lung Carcinoma

The current study aimed to determine if stroma was associated with H&E-stained tissue slices taken from SQCLC samples. Routine H&E-stained slides from the primary tumors were analyzed for the existence of matrix involvement (magnification, x4 and x10). TSR was evaluated on one section derived from the aggressive part of the tumor. Assessment of the TSR was performed in all tumors. As evaluated by two independent research workers (Yue Zhang and Fuman Wang), a total of 50 tumors were indicated to be stroma-poor, and 36 were indicated to be stroma-rich, and controversial outcomes were adjudicated by a subsequent observer (Yifan Chi). Cohen's *κ* indicated a moderate agreement (*κ* = 0.48). The moderate *κ* (0.48) may reflect SQCLC's heterogeneity. However, our adjudication process (third pathologist review) ensured final consensus aligned with clinical outcomes [13].

### 3.3. Correlation of TSR With Other Prognostic Factors

Table 1 demonstrates patient, tumor, and treatment features for the stroma-rich and the stroma-poor groups. Follow up was complete. According to statistical analysis, the 5-year OS rate and no distant metastasis survival rate were 64% and 46%, respectively, in the stroma-poor group and 44% and 31%, respectively, in the stroma-rich group. Median OS for patients in the stroma-poor group was 54 months compared with 39 months for patients in the stroma-rich group. The survival curve and risk curve are shown in Figure 3. There were significant differences in the survival curve and risk curve between the two groups of patients.

In the Cox single-variable and multivariable analysis of OS, the HRs of TSR were 1.91 (95% confidence interval: 1.32–2.95; *p*=0.001) and 2.41 (95% confidence interval: 1.77–3.49; *p* < 0.001). In the Cox single-variable model, TSR, pTNM stage, pT status, pN status, and tumor differentiation were significantly related to OS. In the Cox single-variable and multivariable analysis of no distant metastasis survival, the hazard ratios for TSR were 1.97 (95% confidence interval: 1.34–2.92; *p*=0.001) and 2.27 (95% confidence interval: 1.44–3.51; *p* < 0.001). In the Cox multivariate analysis, TSR, differentiation class, and pathological tumor-node-metastasis stage were indicated to be associated with no distant metastasis survival. So, TSR is an independent prognostic factor for survival and distant metastasis in patients with SQCLC following lung resection (Tables 2 and 3).

## 4. Discussion

Lung cancer is one of the leading causes of cancer-related deaths worldwide. Surgical resection remains the most effective treatment for early-stage NSCLC, with curative intent [14]. However, distant metastasis after surgical resection remains a major concern, as it significantly impacts patient prognosis [15]. Identifying independent prognostic factors that can predict distant metastasis and prognosis is crucial for guiding treatment decisions and improving patient outcomes. While TSR has been studied in gastrointestinal cancers, its prognostic role in SQCLC remains underexplored. Our study is the first to demonstrate that TSR independently predicts distant metastasis in SQCLC post-resection (HR = 2.27, *p* < 0.001), even after adjusting for pTNM stage (Table 3). This complements prior work by Smit et al. [13] but with a longer follow-up (median 69 months) and standardized TSR scoring.

In our study, we investigated the TSR as a potential independent prognostic factor of distant metastasis and prognosis in patients with SQCLC after lung resection. The TSR, which represents the proportion of tumor stroma to tumor parenchyma, has been recognized as an important histopathological parameter reflecting the tumor microenvironment and biological behavior in various cancer types [16].

However, there are only a few studies assessing the relationship between TSR and SQCLC. Therefore, this was the focus of the current study. The optimal threshold standard of TSR was decided on the basis of a maximum discriminability for entire survival and no distant metastasis survival. The 50% cutoff value was typical [17]. In the present study, it was indicated that the 5-year survival ratio and no distant metastasis survival ratio were 64% and 46% in the stroma-poor group, while the values were 44% and 31% in the stroma-rich group.

It is well known that a number of factors are associated with the prognosis of SQCLC [18]. The results of the current study indicated that the TSR was not associated with gender, age, smoking history, drinking history, differentiation class, adjuvant therapy, or pathological tumor-node-metastasis stage. However, the TSR, differentiation grade, pT status, pN status, and pTNM stage were significantly associated with OS and no distant metastasis survival in the single-variable survival analyses, and TSR was also indicated to be an independent prognostic element in multivariable analysis. These results revealed that increased stromal proportion in SQCLC was closely associated with poor outcomes and risks [19].

The underlying mechanisms for the association between TSR and distant metastasis in SQCLC patients are not fully understood [20]. It is possible that the tumor stroma plays a critical role in promoting tumor progression and metastasis through its involvement in angiogenesis, immune cell infiltration, and ECM remodeling [21]. Further studies are needed to elucidate the specific mechanisms by which the tumor stroma influences distant metastasis in SQCLC.

The identification of TSR as an independent prognostic factor of distant metastasis and prognosis in SQCLC patients has significant clinical implications. Firstly, it provides additional information for risk stratification and personalized treatment planning [22]. Patients with a high TSR could be considered for more aggressive adjuvant therapies or targeted therapeutic approaches aimed at reducing the likelihood of distant metastasis. Secondly, TSR could serve as a potential therapeutic target. Modulating the tumor stroma using anti-stromal agents or immunotherapies may offer a novel approach to inhibit distant metastasis and improve patient outcomes [23].

It is important to acknowledge some limitations of our study. Firstly, our study was retrospective in nature and included a relatively small sample size. Prospective and multicenter studies with larger cohorts are warranted to validate our findings. Secondly, other factors not evaluated in our study, such as genetic alterations and molecular markers, may also contribute to distant metastasis and prognosis in SQCLC patients.

Overall, our study provides evidence that the TSR is an independent prognostic factor of distant metastasis and prognosis in patients with SQCLC after lung resection. Further studies are needed to validate these findings and explore the underlying mechanisms. The incorporation of TSR into clinical practice could improve risk stratification and guide treatment decisions for SQCLC patients.

## Figures and Tables

**Table 1:** Clinicopathological characteristics of 86 patients with squamous cell lung carcinoma.

Characteristics	Total (*N* = 86)	Stroma-poor (*n* = 50)	Stroma-rich (*n* = 36)	*p*
	No (%)	No (%)	No (%)	
Gender				0.892
Women	10 11.6	6 60.0	4 40.0	
Men	76 88.4	44 57.9	32 42.1	
Age				0.937
< 60	34 39.5	19 55.9	15 44.1	
≥ 60	52 60.5	31 59.6	21 40.4	
Smoking history				0.796
< 20 P.Y	29 33.7	18 62.1	11 37.9	
≥ 20 P.Y	57 66.3	32 56.1	25 43.9	
Drinking history				0.833
No	34 39.5	20 58.8	14 41.2	
Yes	52 60.5	30 57.7	22 42.3	
Differentiation grade				0.347
Well	50 58.1	31 62.0	19 38.0	
Moderate	28 32.6	15 53.6	13 46.4	
Poor	8 9.3	4 50.0	4 50.0	
pT status				0.453
pT1	26 30.2	16 61.5	10 38.5	
pT2	52 60.5	31 59.6	21 40.4	
pT3	8 9.3	3 37.5	5 62.5	
pN status				0.277
pN0	43 50.0	27 62.8	16 37.2	
pN1	25 29.1	14 56.0	11 44.0	
pN2	14 16.3	8 57.1	6 42.9	
pN3	4 4.6	1 25.0	3 75.0	
pTNM stage				0.131
I	39 45.3	25 64.1	14 35.9	
II	30 34.9	17 56.7	13 43.3	
III	17 19.8	8 47.1	9 52.9	
Adjuvant therapy				0.913
No	38 43.8	22 57.9	16 42.1	
Yes	48 56.2	28 58.3	20 41.7	

*Note:* pT, pathological tumor stage; pN, pathological node stage; pTNM, tumor node metastasis. *p* < 0.05 was considered significant.

Abbreviation: P.Y, package year.

**Table 2:** Cox univariate analysis for survival in 86 patients of squamous cell lung carcinoma.

	Univariate analysis			
	Overall survival		No distant metastasis survival	
	HR (95% CI)	*p*	HR (95% CI)	*p*
Gender		0.703		0.782
Women	1.000 ref	—	1.000 ref	—
Men	1.17 (0.54–2.08)	0.703	1.51 (0.55–2.28)	0.782
Age		0.252		0.288
< 60	1.000 ref	—	1.000 ref	—
≥ 60	1.01 (0.89–1.93)	0.252	1.29 (0.89–1.95)	0.288
Smoking history		0.482		0.577
< 20 P.Y	1.000 ref	—	1.000 ref	—
≥ 20 P.Y	0.88 (0.52–1.38)	0.482	0.93 (0.58–1.47)	0.577
Drinking history		0.266		0.278
No	1.000 ref	—	1.000 ref	—
Yes	0.78 (0.52–1.19)	0.266	0.82 (0.52–1.21)	0.278
Differentiation grade		**< 0.001**		**< 0.001**
Well	1.000 ref	—	1.000 ref	—
Moderate	5.68 (3.51–9.19)	< 0.001	5.69 (3.68–9.68)	< 0.001
Poor	8.79 (4.51–16.93)	< 0.001	9.59 (4.94–18.74)	< 0.001
pT status		**0.004**		**0.003**
pT1	1.000 ref	—	1.000 ref	—
pT2	0.96 (0.98–1.57)	0.982	0.97 (0.64–1.56)	0.944
pT3	2.66 (1.39–5.11)	0.004	2.814 (1.44–5.41)	0.003
pN status		**< 0.001**		**< 0.001**
pN0	1.000 ref	—	1.000 ref	—
pN1	5.43 (2.68–7.50)	< 0.001	5.62 (2.77–7.84)	< 0.001
pN2	14.16 (8.89–29.60)	< 0.001	14.91 (9.14–0.31.23)	< 0.001
pN3	58.59 (23.90–63.18)	< 0.001	60.54 (23.97–73.72)	< 0.001
pTNM stage		**< 0.001**		**< 0.001**
I	1.000 ref	—	1.000 ref	—
II	4.19 (2.44–7.26)	< 0.001	4.37 (2.57–7.57)	< 0.001
III	19.23 (10.57–35.06)	< 0.001	21.88 (11.77–40.48)	< 0.001
Adjuvant therapy		0.824		0.824
No	1.000 ref	—	1.000 ref	—
Yes	0.97 (0.68–1.47)	0.824	0.98 (0.61–1.48)	0.824
TSR		**0.001**		**0.001**
Stroma-poor	1.000 ref	—	1.000 ref	—
Stroma-rich	1.91 (1.32–2.95)	0.001	1.97 (1.34–2.92)	0.001

*Note:* pT, pathological tumor stage; pN, pathological node stage; pTNM, tumor node metastasis. Analysis was performed using the Cox proportion hazard model. *p* < 0.05 was considered significant (indicated in bold).

Abbreviation: TSR, tumor-stroma ratio.

**Table 3:** Cox multivariate analysis for survival in 86 patients of squamous cell lung carcinoma.

	Multivariate analysis			
	Overall survival		No distant metastasis survival	
	HR (95% CI)	*p*	HR (95% CI)	*p*
Differentiation grade	2.41 (1.77–3.49)	< 0.001	2.54 (1.83–3.58)	< 0.001
pT status	0.91 (0.66–1.44)	0.864	0.95 (0.62–1.48)	0.826
pN status	2.89 (1.54–5.33)	0.001	2.67 (1.46–4.82)	0.002
pTNM stage	1.34 (0.62–2.83)	0.435	1.60 (0.77–3.45)	0.242
TSR	2.41 (1.55–3.72)	< 0.001	2.27 (1.44–3.51)	< 0.001

*Note:* pT, pathological tumor stage; pN, pathological node stage; pTNM, tumor node metastasis; Ref., reference. Analysis was performed using the Cox proportion hazard model. *p* < 0.05 was considered significant.

Abbreviations: CI, confidence interval; HR, hazard ratio; TSR, tumor-stroma ratio.

## Data Availability

Data will be made available on request.
